# Supplementary material for: Effect of Ultraviolet Radiation on Reducing Airborne Escherichia coli Carried by Poultry Litter Particles
Source: Animals (Basel). 2022 Nov 16;12(22):3170. doi: 10.3390/ani12223170 (PMC9686630; doi:10.3390/ani12223170)
Supplement: Supplementary file 1 [file animals-12-03170-s001.zip › Supplemental Materials/Table S1, Table S2.pdf]

Table S1. Concentration of airborne *E. coli* without UV irradiance treatment.

Contact Times (s, mean ± SD)	*E. coli* Concentration in Upstream Chamber ($\log_{10}$ CFU m^{-3}, mean ± SD)	*E. coli* Concentration in Downstream Chamber ($\log_{10}$ CFU m^{-3}, mean ± SD)
5.62 ± 0.91	7.1 ± 0.2	6.3 ± 0.7
1.2 ± 0.06	6.6 ± 0.1	5.9 ± 0.1
0.34 ± 0.01	6.5 ± 0.6	5.6 ± 0.3
0.23 ± 0.01	6.2 ± 0.7	5.2 ± 0.1

Table S2. Concentration of airborne *E. coli* with UV irradiance treatment.

Contact Times (s, mean ± SD)	Number of UV Lamps	*E. coli* Concentration in Upstream Chamber (log_{10} CFU m^{-3}, mean ± SD)	*E. coli* Concentration in Downstream Chamber (log_{10} CFU m^{-3}, mean ± SD)
5.62 ± 0.91	1	7.0 ± 0.3	3.4 ± 0.3
	2	7.1 ± 0.2	3.0 ± 0.5
1.2 ± 0.06	1	7.0 ± 0.0	5.0 ± 0.0
	2	6.8 ± 0.1	4.6 ± 0.1
0.34 ± 0.01	1	6.9 ± 0.1	5.0 ± 0.1
	2	6.6 ± 0.2	4.5 ± 0.2
0.23 ± 0.01	1	6.6 ± 0.1	5.2 ± 0.0
	2	6.2 ± 0.3	4.6 ± 0.2
